# Priapism following a juvenile Russell’s viper bite: An unusual case report

**DOI:** 10.1371/journal.pntd.0009242

**Published:** 2021-03-25

**Authors:** Subramanian Senthilkumaran, Harry F. Williams, Ketan Patel, Steven A. Trim, Ponniah Thirumalaikolundusubramanian, Sakthivel Vaiyapuri

**Affiliations:** 1 Manian Medical Centre, Erode, Tamil Nadu, India; 2 Toxiven Biotech Private Limited, Coimbatore, Tamil Nadu, India; 3 School of Biological Sciences, University of Reading, Reading, United Kingdom; 4 Venomtech Limited, Sandwich, United Kingdom; 5 Trichy SRM Medical College Hospital & Research Centre, Trichy, Tamil Nadu, India; 6 School of Pharmacy, University of Reading, Reading, United Kingdom; Texas A&M University Kingsville, UNITED STATES

## Abstract

Following a bite from a juvenile Russell’s viper (*Daboia russelii*), a priapism (painful erection) developed rapidly in a 16-year-old male and only subsided after administration of antivenom 3 hours later. Potential mechanisms for this snakebite-induced priapism are unclear but likely due to venom toxins causing nitric oxide (NO) release and subsequent vasodilation of endothelium in the corpus cavernosum, although the possible involvement of other mechanisms cannot be ruled out. We strongly believe that this unusual case report may lead to further scientific research in order to improve the clinical understanding of the pathophysiology of envenomation due to Russell’s viper bites. Although it is too early to speculate, further research may also discover the possibilities of developing venom-based candidate molecules to treat sexual dysfunction in males and females.

## Introduction

Snakebite envenomation (SBE) results in around 140,000 deaths and over 500,000 permanent disabilities globally every year [1,2]. There are over 600 species of venomous snakes worldwide, and they are classified in to 4 major families [3]. Such a wide range of species are associated with a broad spectrum of envenomation effects such as myonecrosis, coagulopathy, flaccid paralysis, acute kidney injury, and post-traumatic stress disorder [4,5]. These pathologies are generally attributed to the functions of diverse venom components from different families of snakes. Here, we report an unusual case of an adolescent male suffering a priapism following a bite from a juvenile Russell’s viper (*Daboia russelii*). The history of priapism following venomous bites is primarily limited to spiders, particularly, the Brazilian wandering spider (*Phoneutria nigriventer*), where injection of the venom in male mice caused a sustained priapism, frequently before death [6], although examples involving other spiders as well as various other venomous invertebrates are available [7–9]. To our knowledge, priapism following bites from vertebrates (specifically snakes) currently appears to be not documented in the literature. Hence, we report this unusual case of priapism following a Russell’s viper bite and propose the possible mechanisms behind this issue.

## Case presentation

**Fig 1:**
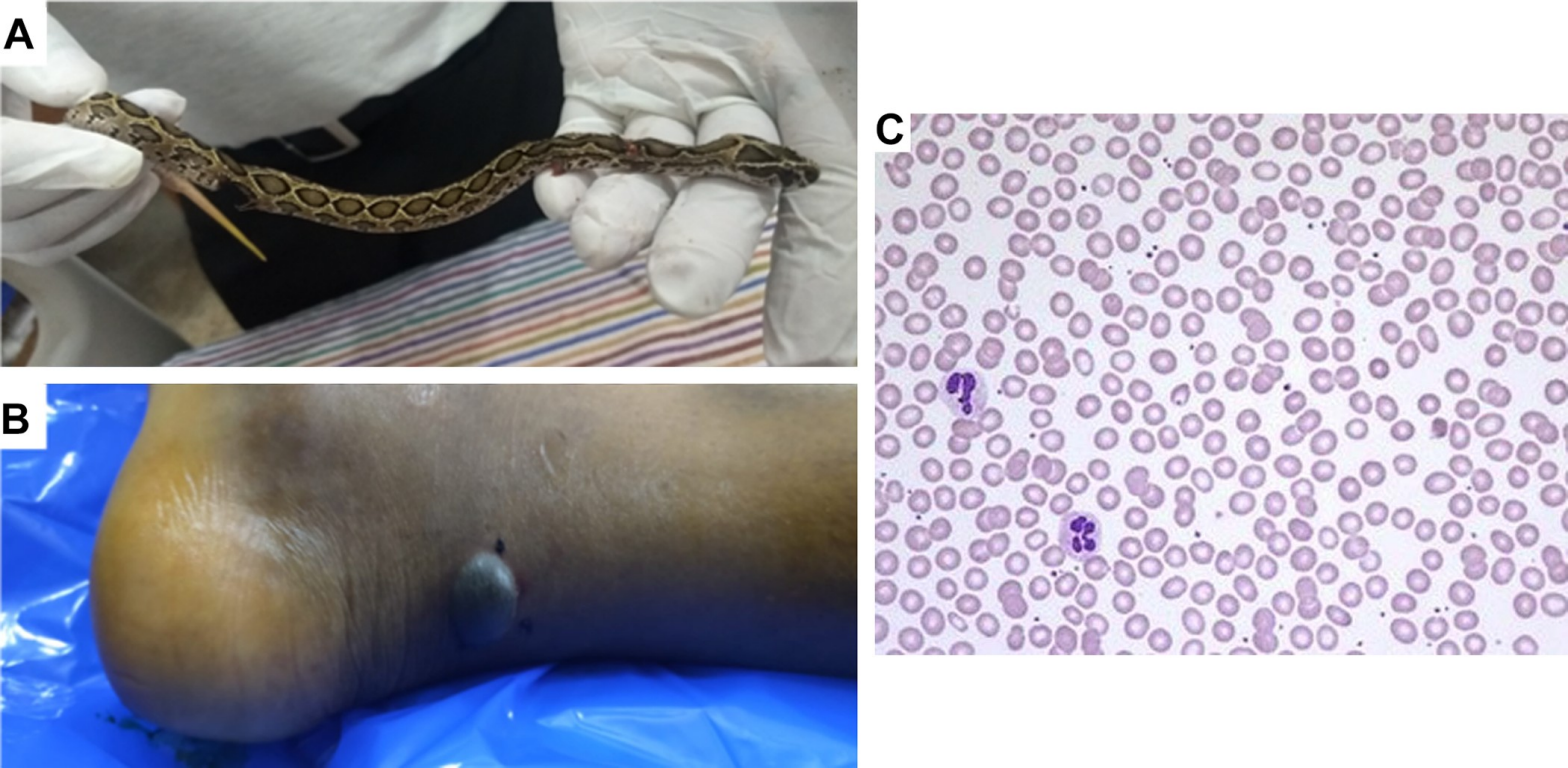
(**A**) The offending snake identified as a juvenile Russell’s viper (*Daboia russelii*) by a herpetologist. (**B**) Blistering below the bite site on the right foot of the victim. (**C**) Microscopic examination of blood smear of the victim shows no significant changes in red blood cells.

A 16-year-old male was brought to the emergency department at around 9:00 pm India Standard Time (IST) presenting a snakebite which he received on his right foot while walking in his garden at around 6:30 pm IST. The offending snake was killed and presented to a qualified herpetologist, who identified it as a juvenile Russell’s viper (Fig 1A). On examination, the victim’s vital signs were within the normal limits. He was conscious, well oriented, anxious, afebrile, and hemodynamically stable. His systemic examination was unremarkable, and local examination revealed swelling of the foot with fang marks, blistering (Fig 1B), and palpable tender right inguinal lymph nodes. Genital examination revealed an engorged, oedematous, and erect penis with tense and tender corpus cavernosa sparing the corpus spongiosum and glans. There was no colour change to the penis. The testis and the prostate were appeared to be normal. The patient stated that he developed spontaneous penile erection within 15 minutes following the snakebite, and the penis had become unusually erect in a way unfamiliar to the victim. He also stated that he had noticed some discomfort during this erection. He denied any history of genital stimulation or desire for sex or similar experience earlier. His erection has continued for the next 2 hours until he received the antivenom. He has no comorbidities and was not on any medication or an abuser of drugs. There was no history of injury to his perineal area, and he had not used any traditional medicines. There were no markers of autonomic manifestations such as excessive sweating, postural hypotension, or piloerection. His prothrombin time and activated partial thromboplastin time were prolonged. The other haematological, metabolic, and biochemical parameters were within the normal limits (Table 1). The results of urinalysis were found to be not related to this condition. There were no dysmorphic red blood cells observed on microscopic examination (Fig 1C). A screening examination for malaria parasite and sickle solubility test were both negative. The victim has received 100 mL of polyvalent (against the “Big Four” snakes of India; Russell’s viper, cobra, saw-scaled viper, and krait) antivenom (Bharat Serums and Vaccines, India) according to the standard protocol, due to his pathological coagulation profile. His priapism gradually resolved, and total detumescence occurred within 60 minutes following initial administration of antivenom. Overall, the priapism lasted for around 3 hours. He received further 100 mL of antivenom to normalise his coagulation profile over the next 24 hours. During the rest of his stay in the hospital, he had normal early morning erections with no further episodes of priapism. He was discharged on the fifth day following snakebite in a stable condition with normal vital signs and coagulation profile. During the weekly follow-up over a period of 4 weeks, he was found to be stable, and his early morning erections were normal. There was no evidence of post-traumatic stress disorder or any other psychiatric morbidity on this patient.

**Table 1:** Basic laboratory examination results of the victim.

Investigation	Results	Unit	Normal range
Haemoglobin	14.1	gms%	13.0–16.0
Total RBC count	4.61	millions/μL	4.00–5.00
HCT	42.3	%	41.00–50.00
MCV	91.8	fl	81.10–96.00
MCH	30.6	pg	27.20–33.20
MCHC	33.3	%	32–36
Total WBC count	19.6	×10^3^ cells/μL	4.00–11.00
Neutrophils	16.13	×10^3^ cells/μL	2.0–7.0
Lymphocytes	2.17	×10^3^ cells/μL	1.0–3.0
Monocytes	1.09	×10^3^ cells/μL	0.1–08
Eosinophils	0.16	×10^3^ cells/μL	0.02–0.5
Basophils	0.05	×10^3^ cells/μL	0.02–0.1
Platelet count	316	×10^3^ cells/μL	150–450
MPV	9.7	fl	6.5–12.0
PDW	10.2	fl	9.0–13.0
Prothrombin time	19.0	Seconds	11.5–16.0

HCT, haematocrit; MCH, mean corpuscular haemoglobin; MCHC, mean corpuscular haemoglobin concentration; MCV, mean corpuscular volume; MPV, mean platelet volume; PDW, platelet distribution width; RBC, red blood cell; WBC, white blood cell.

## Case discussion

Priapism is unpredictable and rarely encountered in clinical practice due to increased (nonischaemic) or decreased (ischaemic) blood flow to penis [10]. Development of penile erection following envenomation and its gradual detumescence following antivenom administration clinically denote that 1 or more components of the venom is likely to have caused the priapism in this victim. Penile erections following exposure to scorpion, widow spider, and box jellyfish venoms have been documented in clinical cases and experimental models, where mechanisms are being investigated [9]. Arachnid envenomation appears to be due to delayed inactivation of sodium channels and downstream calcium flux in corpus cavernosum tissue leading to activation of nitric oxide (NO) synthase (NOS) [9,11,12]. The possible mechanism for penile erection following Russell’s viper envenomation may be due to 1 or more toxins of this venom having an effect on molecules such as NO [13], phosphodiesterases (PDE) [14], ion channels, and/or N-methyl-d-aspartate (NMDA) [15] receptors in penile nitrergic nerves present in human corpus cavernosa as they have been shown to play significant roles in the regulation of erection. However, as venoms from juvenile Russell’s vipers display reduced expression of proteins [16] and defibrinating enzymes such as ancrod and batroxobin from other vipers have been proposed to treat priapism in humans [17,18], it might be possible that the toxins responsible for this effect may be counteracted in older snakes or only expressed in venoms of juvenile snakes. An imbalance of polyamines in snake venoms [19] might also be responsible for priapism if this effect is mediated through NMDA receptors. Hypopituitarism is also a rare, but well documented, complication of Russell’s viper envenomation affecting the sexual organs via pituitary insufficiency [20]. Since Russell’s viper venom has neurotoxic manifestations, it is worth considering the action of this venom via brain synaptosomes to demonstrate if this unusual event may be linked with the rare effects on the pituitary gland or peripheral tissues. It is noteworthy that the venom proteins may largely act on the peripheral tissues as they don’t usually penetrate the blood–brain barrier [21] except some toxins (e.g., apamin from bee venom) that were reported to pass through this barrier [21,22]. Other lines of enquiry resulting from some priapism case studies suggest α2δ1 subunits of voltage-gated calcium channels [23], the target of pregabalin as responsible for this condition. An equine veterinary case suggests a molecular link between the pituitary pars intermedia and sexual dysfunction [24]. Description of a protein complex within the venom of Russell’s viper with reproductive toxicity has been described, and increased NO was noted in the testis of dosed mice, although priapism was absent [25]. NO has long been known to cause relaxation of the corpus cavernosa and is proposed as the mechanism of priapism following envenomation from *Tityus serrulatus* [26]. Further studies into the action of venoms on pathways such as NO and ion channels might also lead to insights into female sexual dysfunction as the mechanisms are largely shared [27], but so far, this has not been reported in SBE victims. Indeed, female sexual dysfunction is an underdiagnosed and therefore, an undertreated condition which needs significant attention in scientific research [28]. Hence, investigating the impact of venom toxins in this context may not only demonstrate the pharmacological actions behind this unusual envenomation effect of Russell’s viper bite but may also reveal potential therapeutic avenues for sexual dysfunction in both males and females.

Although it is too early to speculate the impact of specific venom toxins on sexual dysfunction, this rare case of priapism following a snakebite may point towards further research to determine the beneficial effects of Russell’s viper venom toxins for erectile dysfunction (ED). Given that 90% of men over the age of 80 experience dissatisfaction with their sexual function, ED treatments such as PDE5 inhibitors [sildenafil citrate (e.g., viagra, taladafil, and vardenafil)] are highly commercially viable with ongoing research for better alternatives. When NO is released from endothelial cells, as a result of sexual stimulation, it activates soluble guanylate cyclase and consequently causes an increase in cyclic guanosine monophosphate (cGMP), which relaxes smooth muscle, leading to vasodilation and increased blood flow: an erection. These treatments for ED protect cGMP from degradation by inhibiting PDE5 [9] and therefore rely on NO-relaxing nerve fibres as well as corpus cavernous endothelium which limits the number of patients who are able to benefit from such drugs [10]. Therefore, the use of naturally evolved venom toxins might provide better alternatives for sexual dysfunction without undesired side effects. For example, studies involving toxins from the venom of *P*. *nigriventer* have shown these molecules to provide a promising alternative, and synthetic analogues of the PnTx2-6 have removed side effects such as pain and brain edema [29]. Moreover, these molecules act via a decrease in voltage-gated Na+ channel (Na_v_) inactivation rates [30] could provide an alternative to PDE5 inhibitors. Currently, there are no known relationships between the venom of Russell’s viper and Na_V_ channel activation. However, Na_V_ channel activators are known from other snake venoms [31]. Interestingly, Russell’s viper venom causes vasodilation through voltage and calcium activated potassium channels K_v_ and K_Ca_ [32], hence this possible mechanism can be further investigated for novel ED treatments. In addition, many snake venoms including from Russell’s viper contain PDEs [33]. The presence of both venom enzymes and their inhibitors are known from the same venom, and this may potentially to protect the host from their own venom. It is therefore possible that PDE inhibitors are present in Russell’s viper venom in addition to PDEs, and these may be out of balance in juvenile snakes.

Unusual cases, such as this, are starting points for unique research to determine the unprecedented mechanisms of venom toxins which may lead to better understanding of the envenomation pathology. At this stage, it is unclear what the causative agent is from Russell’s viper venom to induce priapism and what the evolutionary benefit of such a toxin or if it is a rare response from the patient’s own physiology. Indeed, priapism is known to be induced by various factors such as sickle cell disease in men [34], the long-term use of certain drugs [35], vasculitis [36], and trauma (e.g., due to rigorous bicycle riding) [37]. Therefore, further research is required to determine the effects of venoms specifically from Russell’s viper in inducing priapism. Although this case report does not answer plethora of questions concerning the molecular relationships between snake venom toxins and priapism, the data presented here initiate a new line of research in this highly significant area. Notably, as SBE is common in rural tropical areas, medical practitioners have to be primed to look for unusual complications such as this that can be used both diagnostically and in leading future research. This report also reinforces the need to report unusual clinical cases of both SBE and SBE-induced priapism to determine if this effect is an anomaly or connected with a particular subpopulation of Russell’s viper. This clinical case may provide novel platforms for the discovery of new therapeutic agents, research tools, or diagnostics and also demonstrate the importance of attaining a full understanding of envenomation effects to develop improved treatment strategies for SBE.

### Ethical statement

The data collection, consent form, and information sheets were approved by the Institutional Ethics Committee at Toxiven Biotech, Tamil Nadu, India (Reference number: ICMR-Toxiven Ethics 2019–001/002). A written consent was obtained from the victim’s parents (as the victim was a minor) to collect and publish the data presented in this article.
